# Comparative study of statistical methods for detecting association with rare variants in exome-resequencing data

**DOI:** 10.1186/1753-6561-5-S9-S33

**Published:** 2011-11-29

**Authors:** Mohamad Saad, Aude Saint Pierre, Nora Bohossian, Matthias Macé, Maria Martinez

**Affiliations:** 1 INSERM UMR1043, CPTP, CHU Purpan, Toulouse, 31024, France; 2 Université Paul Sabatier, Toulouse, France

## Abstract

Genome-wide association studies for complex traits are based on the common disease/common variant (CDCV) and common disease/rare variant (CDRV) assumptions. Under the CDCV hypothesis, classical genome-wide association studies using single-marker tests are powerful in detecting common susceptibility variants, but under the CDRV hypothesis they are not as powerful. Several methods have been recently proposed to detect association with multiple rare variants collectively in a functional unit such as a gene. In this paper, we compare the relative performance of several of these methods on the Genetic Analysis Workshop 17 data. We evaluate these methods using the unrelated individual and family data sets. Association was tested using 200 replicates for the quantitative trait Q1. Although in these data the power to detect association is often low, our results show that collapsing methods are promising tools. However, we faced the challenge of assessing the proper type I error to validate our power comparisons. We observed that the type I error rate was not well controlled; however, we did not find a general trend specific to each method. Each method can be conservative or nonconservative depending on the studied gene. Our results also suggest that collapsing and the single-locus association approaches may not be affected to the same extent by population stratification. This deserves further investigation.

## Background

Classical genome-wide association studies have successfully detected many common genetic variants that are associated with complex traits. It is likely that low-frequency or rare variants are also contributing to genetic risk [1]. The statistical power to detect phenotypic association with such variants is limited because of the small number of observations for any given variant and a more stringent multiple test correction compared to common variants [2]. The simultaneous analysis of rare variants aims to identify accumulations of minor alleles within the same functional unit (e.g., gene).

Several new methods have been recently proposed to tackle the rare variant problem [2-6]. The principal difference between them lies in the way the information on the multiple rare variants is used. Some methods use a subset of variants that satisfy predefined selection criteria, whereas other methods use all variants. The methods also differ in the way in which the cumulative information on minor alleles within a functional unit is coded. Finally, multivariate collapsing approaches have also been proposed. Most of these recent developments have been applied to association analyses in data from unrelated individuals. A new method has been recently developed [4,6] that can be applied to both unrelated individual and family data.

In this paper, we evaluate and compare the power of different collapsing methods for detecting association of multiple rare variants with a quantitative trait. We first focus on the unrelated individuals data and then incorporate some of these approaches within the general framework of the mixed model for association analysis in the family data set of Genetic Analysis Workshop 17 (GAW17). We tried to answer the following questions: Does the use of a subset of rare variants perform better than using all variants? Do the collapsing approaches perform similarly with unrelated individual and family data sets? The analyses were performed using the GAW17 data with knowledge of the answers [7].

## Methods

We studied the quantitative trait Q1 influenced by 39 variants in nine independent genes.

### Statistical association analysis of rare variants

We carry out the association test at the gene level. Assume that a gene *G* contains *J_G_* variants denoted *SNP^j^*, *j* = 1, …, *J_G_*, and that *MAF_j_* is the minor allele frequency of *SNP^j^*. Let *Y* = (*y*_1_, …, *y_N_*) be the observations of the phenotype Q1 in *N* unrelated individuals, and let *X_iG_* be the vector of genotypes of the SNPs in gene *G* for individual *i*. The genotypes are coded 0, 1, or 2, depending on the number of minor alleles.

Let *T*_maf_ be a selection criterion on minor allele frequency (MAF) values. The association methods we have investigated vary according to a predefined *T*_maf_ value (i.e., less than 1%, less than 5%, or less than 50%) and on the number of collapsing groups. They are all based on a linear regression modeling the relationship between the trait *Y* and the SNP data within a gene. We briefly review these methods in this Methods section. More details are given by Dering et al. [8].

### Association testing in the unrelated individuals data set: univariate collapsing approaches

The univariate collapsing approaches use only a subset of variants that satisfy the constraint MAF ≤ *T*_maf_, where *T*_maf_ is a predefined selection value.

The first univariate collapsing approach is the collapsing and summation test (CAST). Let *X_iG_*(maf) be the vector of genotype scores of the SNPs with MAF <*T*_maf_, and let *J_G_*(maf) be the length of the vector *X_iG_*(maf). The variable *C* = *C_iG_*(maf) (*i* = 1, …, *N*) denotes the two collapsing strategies that we used: collapsing absence/presence (CA) and collapsing proportion (CP). For the CA strategy:(1)

and for the CP strategy:(2)

Equation (1) is based on the presence or absence of the minor allele at any rare variant in gene *G* within an individual [3]. Equation (2) is based on the proportion of rare variants with MAF ≤ *T*_maf_ at which an individual *i* carries at least one copy of the minor allele [5]. The model is *Y* = *Cβ* + *ε*, where  and σ^2^ is the residual variance.

The effect of *β* can be tested with a likelihood ratio test that follows a chi-square distribution with 1 degree of freedom (df).

The second univariate collapsing method is the variable-threshold (VT) approach [2], which uses the CP approach to collapse rare SNPs with MAF <*T*_maf_ but maximizes the statistic according to *T*_maf_. All *T*_maf_ values observed in the gene *G* are considered*.* For each *T*_maf_, a regression *z*-score is computed. Let *z*_max_ be the maximum *z*-score across all *T*_maf_ values. The test of association is based on *z*_max_, and its statistical significance is evaluated empirically by permutation.

The last univariate collapsing method is the weighted-sum (WS) approach [2], which is a generalization of the binary trait weighted-sum approach proposed by Madsen and Browning [4] for quantitative traits. Under this approach, *T*_maf_ = 0.5 (i.e., all variants in a gene *G* are used). The collapsing variable *C* for subject *i* in the WS approach is given by:(3)

where:(4)

For each gene *G*, a genetic score is calculated as:(5)

The significance of Z_G_ is assessed empirically by permutation.

### Association testing in the unrelated individuals data set: combined multivariate and collapsing approach

The combined multivariate and collapsing (CMC) method originally proposed by Li and Leal [3] uses a multiple regression model that contains the CA method’s collapsing variable of SNPs with MAF <*T*_maf_ = 1% and includes all *k* remaining SNPs, *X_j_*_1,…,_*_k_*, individually.

The multivariate model (denoted here as CMC3) is:(6)

Evidence of association (∃*j*, *β_j_* ≠ 0, *j* = 0, …, *k*) is assessed with the likelihood ratio test, which follows a chi-square distribution with (*k* + 1) df.

Using only the SNPs with MAF ≤ 5%, we extended this model in two ways. In both extensions the multivariate model contains the CA collapsing variable of SNPs with MAF < 1%. In the first variation of this model (denoted CMC1), the multivariate model also contains the CA collapsing variable of the other SNPs (i.e., 1% ≤ MAF ≤ 5%). In contrast, in the second extension (denoted CMC2), the other SNPs are included individually in the multivariate model.

The CMC1 model is then written as:(7)

and the test of association is a likelihood ratio test with 2 df.

The CMC2 model is the same as Eq. (6), where *k* is the number of SNPs and 1% ≤ MAF ≤ 5%. Evidence of association is assessed with the likelihood ratio test with (*k* + 1) df.

### Association testing in the unrelated individuals data set: single-marker test

For comparison purposes, we also carried out a single-locus association test. For a gene *G*, association with each SNP was tested using the likelihood ratio test. For each gene *G*, we obtained *J_G_* likelihood ratio test statistics, each with 1 df. The evidence of association at the gene level was based on the maximum of the *J_G_* likelihood ratio test statistics.

Single-marker (SM) tests were conducted with PLINK, version 1.07 [9]. The R.2.10.1 software was used for all collapsing approaches except the VT and WS approaches. For these two approaches we used the R script (http://genetics.bwh.harvard.edu/vt/dokuwiki/) [2], and we set the number of permutations to 1,000.

### Association testing in the family data set

We used the measured genotype (MG) test [10], which is a linear mixed model:(8)

where:(9)

 and  are the polygenic and the residual variances, respectively, and  is the kinship matrix in family *i*. The SNP data in relatives were collapsed as described under the CA, CP, and WS collapsing approaches. In these three approaches, the test of association is a likelihood ratio test with 1 df. In addition, we also carried out the bivariate CMC1 approach using the likelihood ratio test with 2 df. We could not evaluate the VT approach because it maximizes *T*_maf_. We carried out the MG test using the QTDT software (http://www.sph.umich.edu/csg/abecasis/QTDT/).

### Type I error rate and power estimates

The empirical distribution of each association approach was evaluated in unrelated individuals and in family data. Type I error and power rates were estimated by testing association of Q1 to each of the seven false causal genes and each of the nine true causal genes, respectively, using the 200 replicates. Type I error and power rates were derived at a nominal level of *α* = 5%.

In the unrelated individuals data set, we evaluated association with Q1 using 10 approaches: CA1 and CA5 with *T*_maf_ = 1% and 5%, respectively; CP1 and CP5 with *T*_maf_ = 1% and 5%, respectively; and VT, WS, CMC1, CMC2, CMC3, and SM. For the WS and VT tests, we used empirical *P*-values. For all remaining association tests we used tabulated nominal *P*-values. In each replicate, we tested for association of Q1 with each of the 16 genes using each of the 10 approaches. For each gene and for each association procedure we computed the proportion of replicates having a *P*-value ≤ *α*. For the SM approach, we applied a Bonferroni correction to account for the multiple tests; we computed the proportion of replicates such that the lowest *P*-value out of the *J_G_* SNPs was less than or equal to *α*/*J_G_*.

In the family data set, we evaluated similarly the following five approaches: CA1 and CA5 with *T*_maf_ = 1% and 5%, respectively; CP1 and CP5 with *T*_maf_ = 1% and 5%, respectively; and SM. We also evaluated the WS approach but used the tabulated *P*-value derived from a chi-square distribution with 1 df.

## Results and discussion

The characteristics of the nine causal and seven noncausal genes are shown in Table 1. The total number of SNPs (causal and noncausal) per gene is given along with their distributions by MAF category. The MAF for the causal variants ranges from 0.07% to 16.5% in the 1000 Genomes Project data (for unrelated individuals), and the number of causal variants per gene varies from 1 (*VEGFC*, *VEGFA*) to 11 (*FLT1*). One causal gene (*VEGFC*) has one single SNP, and thus only one association approach (SM) can be applied. For the noncausal genes, the number of SNPs per gene ranges from 6 (*CTSS*) to 83 (*LY75*), and, as for the causal genes, most (>70%) of the SNPs are uncommon (MAF < 5%).

**Table 1:** Characteristics of the studied genes

Chromosome	Gene	*K*	MAF (%)	*V*	*K* (*V*) > 5%	5% >*K* (*V*) > 1%	*K* (*V*) < 1%
Causal genes							
1	*ARNT*	18	0.07; 43.11	5	1 (0)	2 (1)	15 (4)
1	*ELAVL4*	10	0.07; 43.11	2	2 (0)	1 (0)	7 (2)
13	*FLT1*	35	0.07; 29.05	1	3 (1)	7 (2)	25 (8)
5	*FLT4*	10	0.07; 2.08	2	0 (0)	2 (0)	8 (2)
14	*HIF1A*	8	0.07; 1.2	4	0 (0)	1 (1)	7 (3)
19	*HIF3A*	21	0.07; 38.52	3	4 (0)	2 (0)	15 (3)
4	*KDR*	16	0.07; 16.5	10	1 (1)	1 (1)	14 (8)
6	*VEGFA*	6	0.07; 2.37	1	0 (0)	1 (0)	5 (1)
4	*VEGFC*	1	0.07; 0.07	1	0 (0)	0 (0)	1 (1)
Noncausal genes							
1	*PTGFR*	16	0.07; 1.69	0	0	3	13
1	*IFI44*	22	0.07; 11.33	0	1	1	20
1	*FAM73A*	10	0.07; 0.5	0	0	0	10
17	*MAPT*	27	0.07; 35.58	0	5	7	15
1	*CTSS*	6	0.07; 33.28	0	1	1	4
5	*FOXI1*	15	0.07; 37.30	0	2	0	12
2	*LY75*	83	0.07; 45.91	0	11	12	60

*K*, number of variants in gene; *V*, number of true causal variants in gene.

### Estimates of Type I error and power rates in the unrelated individuals data set

Table 2 shows the type I error rates estimated at the gene level of each association approach for the unrelated individuals data set. As can be seen, the type I error rate is not well controlled no matter which association approach is used: The rates can be higher or lower than expected. For some genes, almost all association approaches show inflated type I error rates (e.g., *MAPT*, *IFI44*). Conversely, for some other genes (*FOXI1*, *LY75*), the type I error rates of some approaches are inflated, whereas the other approaches tend to be conservative.

**Table 2:** Type I error rates at *α* = 5% by gene in the unrelated individuals data set

Gene	SM^a^	*T*_maf_ = 0.01		*T*_maf_ = 0.05		WS	VT	CMC1	CMC2	CMC3
							
		CA	CP	CA	CP					
Unadjusted Q1										
*CTSS*	0.020	0.005	0.005	0.030	0.040	0.055	0.020	0.020	0.020	0.030
*FAM73A*	0.020	0.035	0.035	n/a	n/a	0.075	0.055	n/a	n/a	n/a
*FOXI1*	0.150	0.040	0.030	0.040	0.030	0.000	0.000	n/a	n/a	0.110
*PTGFR*	0.040	0.020	0.025	0.025	0.025	0.010	0.080	0.035	0.040	n/a
*IFI44*	0.350	0.055	0.050	0.110	0.140	0.040	0.120	0.305	0.305	0.220
*MAPT*	0.175	0.100	0.200	0.610	0.350	0.555	0.390	0.130	0.110	0.115
*LY75*	0.075	0.010	0.005	0.015	0.030	0.020	0.010	0.065	0.075	0.155
Q1 adjusted for the top five principal components										
*CTSS*	0.015	0.040	0.040	0.040	0.040	0.125	0.060	0.025	0.030	0.045
*FAM73A*	0.025	0.005	0.005	n/a	n/a	0.020	0.020	n/a	n/a	n/a
*FOXI1*	0.040	0.020	0.030	0.020	0.030	0.000	0.000	n/a	n/a	0.035
*PTGFR*	0.015	0.065	0.025	0.010	0.015	0.125	0.060	0.035	0.030	n/a
*IFI44*	0.075	0.030	0.025	0.010	0.015	0.010	0.015	0.020	0.020	0.000
*MAPT*	0.055	0.010	0.015	0.025	0.040	0.010	0.005	0.010	0.050	0.215
*LY75*	0.055	0.005	0.010	0.010	0.010	0.060	0.030	0.015	0.025	0.015

Estimates outside the 95% confidence interval are underlined. n/a, not applicable.

^a^ Bonferroni-corrected *P*-value.

Overall, the SM and CMC3 approaches appear to have inflated type I errors more frequently. Interestingly, these two approaches are the only ones that used the common SNPs individually. Clearly, several SNPs in these sequence data, including those in our noncausal genes, have population-specific allele frequencies. Given that the genotype data were not simulated, we hypothesize that the inflated rates could be explained by the observed differences in the mean of Q1 between the four populations (−0.059, −0.002, 0.021, and 0.072 in Africans, Chinese, Japanese, and Europeans, respectively.

We recomputed the type I error accounting for possible clusters. First, we ran a principal components (PC) analysis with Eigenstrat [11] using the full mini-exome SNP data excluding SNPs with MAF < 5%. In each replicate, we computed the residual of Q1 obtained by regression of Q1 on the first five PCs. We reestimated the type I error levels using the residual of Q1 as the phenotype. The last 10 columns of Table 2 show the results. As can be seen, after adjusting for the five PCs, only a few of the type I error estimates remained higher than expected. In fact, most of the estimates were lower than expected.

In conclusion, to estimate the power of these approaches in the data sets, we used two strategies (Table 3): Power was first computed at a theoretical level of 5%, although the different approaches may not have comparable true false-positive rates; second, power was computed accounting for the five PCs, that is, using the residuals of Q1. All methods performed well for the *KDR* and *FLT1* genes. Conversely, all but two methods performed poorly (power < 10%) for two genes: For *ELAVL4* the power was greater than 30% using the SM and CMC3 approaches, and for *HIF3A* the power was greater than 17% for the CMC2 and CMC3 approaches. For the remaining four genes, one of the pooling methods outperformed the SM method after a Bonferroni correction. In these data, the CA and CP approaches had roughly similar power, and so, in what follows, the CP method will serve as a reference.

**Table 3:** Power rates at *α* = 5% by gene in the unrelated individuals data set

Gene	SM^a^	*T*_maf_ = 0.01		*T*_maf_ = 0.05		WS	VT	CMC1	CMC2	CMC3
							
		CA	CP	CA	CP					
Unadjusted Q1										
*ARNT*	0.86	0.04	0.04	0.79	0.83	0.53	0.76	0.93	0.96	0.94
*ELAVL4*	0.31	0.05	0.05	0.05	0.05	0.00	0.06	0.07	0.07	0.41
*FLT1*	0.99	0.85	0.91	1.00	1.00	1.00	1.00	1.00	1.00	1.00
*FLT4*	0.33	0.41	0.38	0.65	0.62	0.78	0.76	0.50	0.47	n/a
*HIF1A*	0.42	0.07	0.07	0.62	0.59	0.45	0.51	0.62	0.62	n/a
*HIF3A*	0.02	0.03	0.02	0.07	0.07	0.06	0.04	0.20	0.17	0.10
*KDR*	0.96	0.97	0.99	1.00	1.00	1.00	1.00	0.99	0.99	1.00
*VEGFA*	0.26	0.13	0.13	0.41	0.44	0.54	0.45	0.31	0.31	n/a
*VEGFC*	0.58	n/a	n/a	n/a	n/a	n/a	n/a	n/a	n/a	n/a
Q1 adjusted for the top five principal components										
*ARNT*	0.44	0.05	0.05	0.49	0.05	0.37	0.44	0.56	0.67	0.60
*ELAVL4*	0.07	0.07	0.07	0.07	0.07	0.05	0.12	0.06	0.06	0.01
*FLT1*	1.00	0.67	0.80	0.98	1.00	1.00	1.00	0.99	1.00	1.00
*FLT4*	0.09	0.03	0.02	0.04	0.03	0.01	0.02	0.04	0.06	n/a
*HIF1A*	0.13	0.08	0.08	0.00	0.01	0.01	0.01	0.19	0.19	n/a
*HIF3A*	0.03	0.05	0.03	0.01	0.00	0.00	0.00	0.03	0.04	0.03
*KDR*	0.74	0.63	0.74	0.84	0.85	0.99	0.93	0.72	0.69	0.78
*VEGFA*	0.25	0.13	0.13	0.04	0.06	0.19	0.32	0.08	0.10	n/a
*VEGFC*	0.56	n/a	n/a	n/a	n/a	n/a	n/a	n/a	n/a	n/a

Estimates outside the 95% confidence interval are underlined. n/a, not applicable.

^a^ Bonferroni-corrected *P*-value.

The choice of the threshold *T*_maf_ seems to have a large effect on power, and, in general, the power is higher when the criteria are less stringent (*T*_maf_ = 5% vs. 1%). Although this is not surprising for genes with causal SNPs having 1% < MAF < 5% (*ARNT*, *HIF1A*), we made the same observation for genes with all causal SNPs having a MAF < 1% (*FLT4* and *VEGFA*; see Table 1). This may suggest that allele correlation within these genes exists among causal and noncausal rare variants. The VT approach, which does not require a predefined choice on *T*_maf_, did not appear to outperform the CP approach. On the other hand, one of the univariate (WS) or multivariate (CMC3) collapsing methods that uses all SNPs showed better power than the CP method. This again may be explained by allele correlation among SNPs. When adjusting for population stratification, again, all approaches had the greatest power for the *FLT1* and *KDR* genes and the lowest power for the *ELAVL4* and *HIF3A* genes. Nonetheless, most power estimates were lower, and the power drop was noticeable, especially for the *FLT4* and *HIF1A* genes. However, it is unclear whether this drop is fully explained by the lower values of the adjusted false-positive rates.

### Estimates of type I error and power rates in the family data set

Table 4 shows the type I error and power rates estimated at the gene level of each association approach for the family data set. It also shows the number of SNPs, causal and noncausal, that are polymorphic in the family samples. Type I error rates appeared to be better controlled in the family data than in the unrelated individuals data set with a few exceptions, especially the *MAPT* gene, for which most type I errors were biased upward. This gene is located in a genomic region with a low recombination rate and a long range of linkage disequilibrium. All association approaches show high and similar power rates for *VEGFA*. High power (>80%) was observed for *FLT1* using the SM and CP approaches and for *KDR* using the CA(0–5%), CP(0–5%), VT, and CMC1 approaches. In general, as observed in the unrelated individuals data set, the CA and CP approaches showed greater power under the less stringent *T*_maf_ criterion of 5% versus when *T*_maf_ = 1%.

**Table 4:** Type I error and power at *α* = 5% by gene in family data set

Gene	*N*	*N* (*V*) with MAF < 5%	*N* (*V*) with MAF < 1%	SM^a^	*T*_maf_ = 0.01		*T*_maf_ = 0.05		WS	CMC1
							
					CA	CP	CA	CP		
Noncausal genes: type I error										
*PTGFR*	7	4 (0)	7 (0)	0.030	0.095	0.065	0.015	0.010	0.070	0.030
*IFI44*	9	7 (0)	8 (0)	0.060	0.030	0.025	0.030	0.040	0.010	0.175
*FAM73A*	3	3 (0)	3 (0)	0.025	0.020	0.020	0.015	0.020	0.035	n/a
*MAPT*	19	8 (0)	14 (0)	0.210	0.145	0.180	0.035	0.010	0.155	0.015
*CTSS*	3	2 (0)	2 (0)	0.020	0.015	0.015	0.015	0.015	0.020	n/a
*FOXI1*	5	3 (0)	3 (0)	0.020	0.020	0.055	0.055	0.055	0.045	0.000
*LY75*	49	30 (0)	39 (0)	0.055	0.070	0.045	0.030	0.035	0.120	0.035
Causal genes: power										
*ARNT*	7	6 (2)	4 (1)	0.04	0.04	0.03	0.01	0.01	0.12	0.03
*ELAVL4*	8	6 (1)	5 (1)	0.13	0.07	0.07	0.10	0.10	0.04	0.07
*FLT1*	16	13 (4)	8 (2)	0.95	0.02	0.02	0.57	0.82	0.44	0.33
*FLT4*	3	3 (0)	2 (0)	0.04	0.16	0.16	0.17	0.17	0.12	0.10
*HIF1A*	1	1 (1)	0 (0)	0.01	n/a	n/a	0.05	n/a	0.05	n/a
*HIF3A*	12	8 (1)	6 (1)	0.10	0.01	0.01	0.04	0.05	0.13	0.03
*KDR*	5	4 (4)	3 (3)	0.61	0.51	0.51	0.89	0.89	0.91	0.82
*VEGFA*	4	4 (1)	3 (1)	1	1	1	1	1	0.82	1
*VEGFC*	1	1 (1)	1 (1)	1	n/a	n/a	n/a	n/a	n/a	n/a

*N*, number of polymorphic SNPs. *V*, number of polymorphic causal variants.

^a^ Bonferroni-corrected *P*-value.

### Power of collapsing approaches in unrelated individuals versus family data set

Two causal genes (*FLT1*, *KDR*) were consistently detected with good power (>80%) in the unrelated individual and family data sets, irrespective of the association approach. One gene (*VEGFA*) was detected in the family sample but not in the unrelated individuals data set (power < 54%, or power < 32% after adjusting for population stratification). Conversely, *ARNT* was detected in the unrelated individuals data set (power = 96%, or power = 77% after adjusting for population stratification) but not in the family data (power = 12%).

## Conclusions

We found that for some genes collapsing approaches may be powerful tools to detect multiple rare variants for complex traits. In particular, the choice of the threshold *T*_maf_ seems to have a large effect on power, and, in general, we found a higher power when the criterion was less stringent (*T*_maf_ = 5% vs. 1%). In the same vein, including all SNPs, whether by means of a univariate or a multivariate collapsing approach, can improve the power. In addition, a few of the causal genes were detected in both the related and the unrelated individuals data, whereas other causal genes were detected only in either the unrelated individuals or the family data. However, in these data the power of association was often limited. More important, we found that type I error rates may be highly variable between genes and between approaches.

We faced the challenge of assessing the proper type I error to validate our power comparisons. We acknowledge that our type I and type II error rates may not be generalized because of the way the GAW17 data were simulated: Phenotype but not genotype data were generated. Further, because the genotypes of founders did not vary between replicates, each family was either always informative (at least one founder carries a causal variant) or never informative (no founder carries a causal variant) for testing association to a given causal variant.

Finally, our results also raise an interesting point that might deserve future investigation, namely, that the collapsing and the single-locus association approaches may not be affected to the same extent by population stratification. Our results suggest that collapsing approaches may be more robust, especially in the presence of multiple variants.

## Competing interests

The authors declare that there are no competing interests.

## Authors’ contributions

MS, ASP and MMacé performed the statistical analyses. MS, NB, and MMartinez drafted the manuscript. MMartinez conceived the study design and coordinated the study. All authors read and approved the final manuscript.

## References

[B1] Altshuler D Daly MJ Lander ES Genetic mapping in human disease Science 2008 322 881 888 10.1126/science.1156409 18988837 PMC2694957

[B2] Price AL Kryukov GV de Bakker PI Purcell SM Staples J Wei LJ Sunyaev SR Pooled association tests for rare variants in exon-resequencing studies Am J Hum Genet 2010 86 832 838 10.1016/j.ajhg.2010.04.005 20471002 PMC3032073

[B3] Li B Leal SM Methods for detecting associations with rare variants for common diseases: application to analysis of sequence data Am J Hum Genet 2008 83 311 321 10.1016/j.ajhg.2008.06.024 18691683 PMC2842185

[B4] Madsen BE Browning SR A groupwise association test for rare mutations using a weighted sum statistic PLoS Genet 2009 5 e1000384 10.1371/journal.pgen.1000384 19214210 PMC2633048

[B5] Morris AP Zeggini E An evaluation of statistical approaches to rare variant analysis in genetic association studies Genet Epidemiol 2009 34 188 193 10.1002/gepi.20450 PMC2962811 19810025

[B6] Zhu X Feng T Li Y Lu Q Elston RC Detecting rare variants for complex traits using family and unrelated data Genet Epidemiol 2010 34 171 187 10.1002/gepi.20449 19847924 PMC2811752

[B7] Almasy LA Dyer TD Peralta JM Kent JW Jr Charlesworth JC Curran JE Blangero J Genetic Analysis Workshop 17 mini-exome simulation BMC Proc 2011 5 suppl 9 S2 22373155 10.1186/1753-6561-5-S9-S2 PMC3287854

[B8] Dering C Pugh E Ziegler A Statistical analysis of rare sequence variants: an overview of collapsing methods Genet Epidemiol 2011 X suppl X X X 10.1002/gepi.20643 PMC3277891 22128052

[B9] Purcell S Neale B Todd-Brown K Thomas L Ferreira MA Bender D Maller J Sklar P de Bakker PI Daly MJ PLINK: a tool set for whole-genome association and population-based linkage analyses Am J Hum Genet 2007 81 559 575 10.1086/519795 17701901 PMC1950838

[B10] Boerwinkle E Chakraborty R Sing C The use of measured genotype information in the analysis of quantitative phenotypes in man. I. Models and analytical methods Ann Hum Genet 1986 50 181 194 10.1111/j.1469-1809.1986.tb01037.x 3435047

[B11] Price AL Patterson NJ Plenge RM Weinblatt ME Shadick NA Reich D Principal components analysis corrects for stratification in genome-wide association studies Nat Genet 2006 38 904 909 10.1038/ng1847 16862161

